# Vitamin K antagonist use: evidence of the difficulty of achieving and maintaining target INR range and subsequent consequences

**DOI:** 10.1186/s12959-016-0088-y

**Published:** 2016-06-13

**Authors:** Jeff R. Schein, C. Michael White, Winnie W. Nelson, Jeffrey Kluger, Elizabeth S. Mearns, Craig I. Coleman

**Affiliations:** Janssen Scientific Affairs, LLC, Health Economics & Outcomes Research, Raritan, NJ USA; Department of Pharmacy Practice, University of Connecticut School of Pharmacy, 69 N. Eagleville Road, Storrs, CT 06269-3092 USA; Hartford Hospital Division of Cardiology, 80 Seymour Street, Hartford, CT 06102-5037 USA

**Keywords:** Vitamin K antagonists, International normalized ratio, Anticoagulation, Atrial fibrillation, Venous thromboembolism

## Abstract

Vitamin K antagonists (VKAs) are effective oral anticoagulants that are titrated to a narrow therapeutic international normalized ratio (INR) range. We reviewed published literature assessing the impact of INR stability - getting into and staying in target INR range - on outcomes including thrombotic events, major bleeding, and treatment costs, as well as key factors that impact INR stability.

A time in therapeutic range (TTR) of ≥65 % is commonly accepted as the definition of INR stability. In the real-world setting, this is seldom achieved with standard-of-care management, thus increasing the patients’ risks of thrombotic or major bleeding events. There are many factors associated with poor INR control. Being treated in community settings, newly initiated on a VKA, younger in age, or nonadherent to therapy, as well as having polymorphisms of CYP2C9 or VKORC1, or multiple physical or mental co-morbid disease states have been associated with lower TTR. Clinical prediction tools are available, though they can only explain <10 % of the variance behind poor INR control.

Clinicians caring for patients who require anticoagulation are encouraged to intensify diligence in INR management when using VKAs and to consider appropriate use of newer anticoagulants as a therapeutic option.

## Background

Vitamin K antagonists (VKAs) such as warfarin inhibit the enzyme vitamin K epoxide reductase and consequently the recycling of inactive vitamin K epoxide back to its active, reduced form [1]. Vitamin K in its active form is required for the synthesis of various clotting factors (II, VII, IX and X) involved in the coagulation cascade (as well as the anti-clotting proteins C and S); and thus, VKAs result in the depletion of these factors (within 72–96 h after dosing) and an anticoagulated state.

VKAs are indicated for the prevention of thrombotic events in patients with atrial fibrillation (AF) and following venous thromboembolism (VTE) [2, 3]. For stroke prevention in AF patients, VKA therapy that is dose-adjusted to maintain an international normalized ratio (INR) range of 2.0 to 3.0 is associated with a 64 % reduction in the risk of stroke compared to placebo [4]. In patients suffering an acute VTE (either deep vein thrombosis (DVT) or pulmonary embolism (PE)), adjusted-dose VKA use (preceded by a parenteral anticoagulant) significantly reduces the risk of recurrence of thrombotic events [3, 5, 6]. Adjusted-dose VKAs are included in clinical guidelines for AF and VTE [2, 3, 7, 8] with a target INR range of 2.0–3.0.

The objective of this paper is to provide an assessment of “INR stability” with VKA use and patient outcomes in contemporary practice. INR stability refers to achieving and maintaining target INR range (typically 2–3, but not always). Therefore, if target INR range is not achieved or maintained this would be considered INR *in*stability.

We will determine: 1) to what extent INR instability can be anticipated, 2) whether INR instability is predictable, and 3) the consequences of INR instability.

### Metrics of INR

**Fig. 1:**
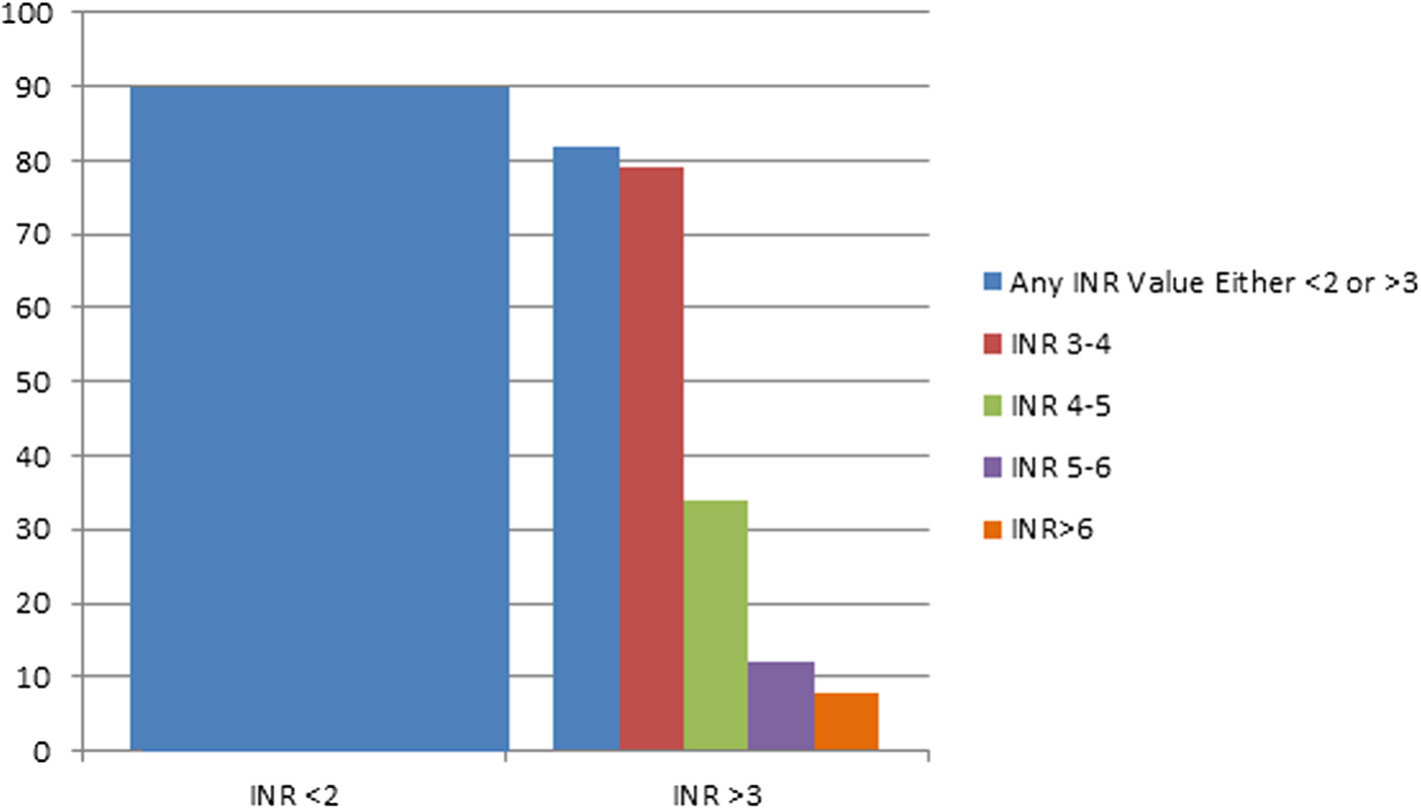
Percent of patients with ≥1 INRs outside the normal therapeutic range. This figure displays the % of people in an analysis [34] of the CoagCheckTM database with at least one INR value outside of the normal therapeutic range with blue boxes showing the percent of patients who were either below 2.0 (90 %) or above 3.0 (82 %). The red, green, purple, and orange boxes display the percent of people who ever achieved a level of 3.0–4.0, 4.0–5.0, 5.0–6.0, and >6.0, respectively. The same individual could be represented in multiple categories given their INRs achieved over time including being below 2.0 and above 3.0

Despite 60 years of clinical experience, the maintenance of stable INR in patients using VKA remains a challenging task. While numerous metrics have been used in clinical studies of VKAs to assess the quality of anticoagulation control [9, 10], time in therapeutic range (TTR) (most commonly calculated using Rosendaal’s method of linear interpolation [11]) is the most frequently reported. Experts have suggested that the minimum target TTR should be no less than 65 % [12–15] but this goal is often not met [16–22] even in modern day RCTs [23–32] (Table 1). A large observational assessment of 40,404 patients in the VA population demonstrated that 42 % of patients had INR stability (defined as TTR > 70 %) while 34 % had moderate instability (TTR 50 to 70 %), and 23 % had high instability (TTR <50 %) [33]. A recently published retrospective analysis from the CoagClinic™ database assessed 9433 patients who met the inclusion criteria and had been using warfarin for over 6 months [34]. In these chronic warfarin patients, more than 90 % had at least one value below 2 and 82 % had at least one value above 3 (Fig. 1).

**Table 1:** Mean time in the therapeutic range observed in recent atrial fibrillation and venous thromboembolism randomized controlled trials of novel target oral anticoagulants

Study	Disease state	Mean TTR	TTR in month 1^a^	TTR in later months^a^
ARISTOTLE	NVAF	62 %		
ENGAGE-TIMI-48 2013	NVAF	65 %		
RE-LY 2009	NVAF	64 %		
ROCKET-AF, 2010	NVAF	55 %		
AMPLIFY	VTE	61 %	NR	NR
EINSTEIN-DVT	DVT	58 %	54 %	66 % (month 10)
EINSTEIN-PE	PE	63 %	58 %	73 % (month 11)
Hokusai-VTE	VTE	64 %	NR	NR
RECOVER 1	VTE	60 %	53 %	66 % (month 6)
RECOVER 2	VTE	57 %	51 %	54–62 % (months 3–6)

*DVT* deep vein thrombosis, *NR* not reported, *NVAF* nonvalvular atrial fibrillation, *PE* pulmonary embolism, *TTR* time in the therapeutic range, *VTE* venous thromboembolism

^a^For venous thromboembolism studies only

Using data from the multicenter ORBIT-AF (Outcomes Registry for Better Informed Treatment of Atrial Fibrillation) registry, the INR stability of 3749 patients on chronic warfarin therapy for 6 months was assessed [35]. Only 26 % (95%CI: 24 to 27 %) of patients had 80 % or more of their INRs between 2 and 3. Among this subgroup with INR stability, 92 % (95%CI: 90 to 94 %) had at least one value outside of the normal INR range while 36 % (95%CI: 33 to 39 %) had an INR below 1.5 or above 4 over the subsequent year. Thus, even the “cream of the crop” – those patients able to achieve most of their values within target range within a 6-month period – had at least occasional out-of-range values over longer-term follow-up.

Multiple meta-analyses of randomized and real-world studies have been performed in order to estimate the quality of INR control in AF and VTE populations receiving VKAs [16–20, 22]. These meta-analyses demonstrate poor INR control to be ‘the rule rather than the exception’ with TTRs and proportion of INR measurement in range typically falling near or below 60 % and nearly twice the amount of time being spent below versus above the therapeutic INR range (Table 2) [16–20, 22].

**Table 2:** Results of meta-analyses evaluating the international normalized ratio stability in atrial fibrillation or venous thromboembolism patients

Meta-Analysis, Year	Population	TTR, % (95 % CI)	TBR, % (95 % CI)	TAR, % (95 % CI)	PINRR, % (95 % CI)	PINRBR, % (95 % CI)	PINRAR, % (95 % CI)
Mearns 2014	AF	61 (59 to 62)	25 (23 to 27)	14 (13 to 15)	59 (53 to 59)	26 (23 to 29)	13 (11 to 17)
Mearns 2014	VTE	61 (59 to 63)	25 (23 to 26)	15 (14 to 17)	59 (54 to 64)	26 (23 to 29)	13 (9 to 19)
Erkens 2012 (Month 1)	VTE	54 (NR)	42 (NR)	12 (NR)	NR	NR	NR
Erkens 2012 (Months 1–3)		56 (NR)	35 (NR)	19 (NR)	NR	NR	NR
Erkens 2012 (Month 1–6+)		60 (NR)	24 (NR)	17 (NR)	NR	NR	NR
Erkens 2012 (Month 4–12+)		75 (NR)	21 (NR)	12 (NR)	NR	NR	NR
Wan 2008 (RCT)	AF	67 (44 to 73)	20 (18 t to 40)	14 (9 to 17)	67 (48 to 84)	24 (14 to 32)	8 (2 to 24)
Wan 2008 (Prospective)		61 (56 to 66)	21 (14 to 29)	14 (13 to 30)	---	---	---
Wan 2008 (Retrospective)		59 (29 to 75)	25 (9 to 52)	14 (9 to 39)	53 (34 to 68)	26 (10 to 51)	17 (14 to 29)
Cios 2009	AF	59 (57 to 61)	NR	NR	NR	NR	NR
Reynolds 2004	AF	61 (NR)	26 (NR)	13 (NR)	61 (NR)	25 (NR)	14 (NR)

*AF* atrial fibrillation, *CI* confidence interval, *NR* not reported, *PINRAR* proportion of INRs above range, *PINRBR* proportion of INRs below range, *PINRR* proportion of INRs in range; *RCT* randomized controlled trial, *TAR* time above range, *TBR* time below range, *TTR* time in range, *VTE* venous thromboembolism

--- = evaluated, but no data

The literature from clinical trials and observational studies substantiate that INR stability is not readily attainable and when it occurs, is rarely sustainable over time.

### Consequences of INR Instability

#### Outcomes

The consequences of INR instability are multifaceted. INR instability was associated with clinical events, higher level of medication non-persistence and discontinuation, utilization of more healthcare resources, and therefore, higher costs. According to meta-analyses of AF or mixed populations assessing INR control and associated events [18, 36–38], greater than half of all thromboembolic events occurred when patients have an INR < 2.0, while over 40 % of all hemorrhagic events occurred at an INR >3.0. In VTE patients, subtherapeutic INRs were found to be present during 58 % of recurrent VTEs [17].

**Fig. 2:**
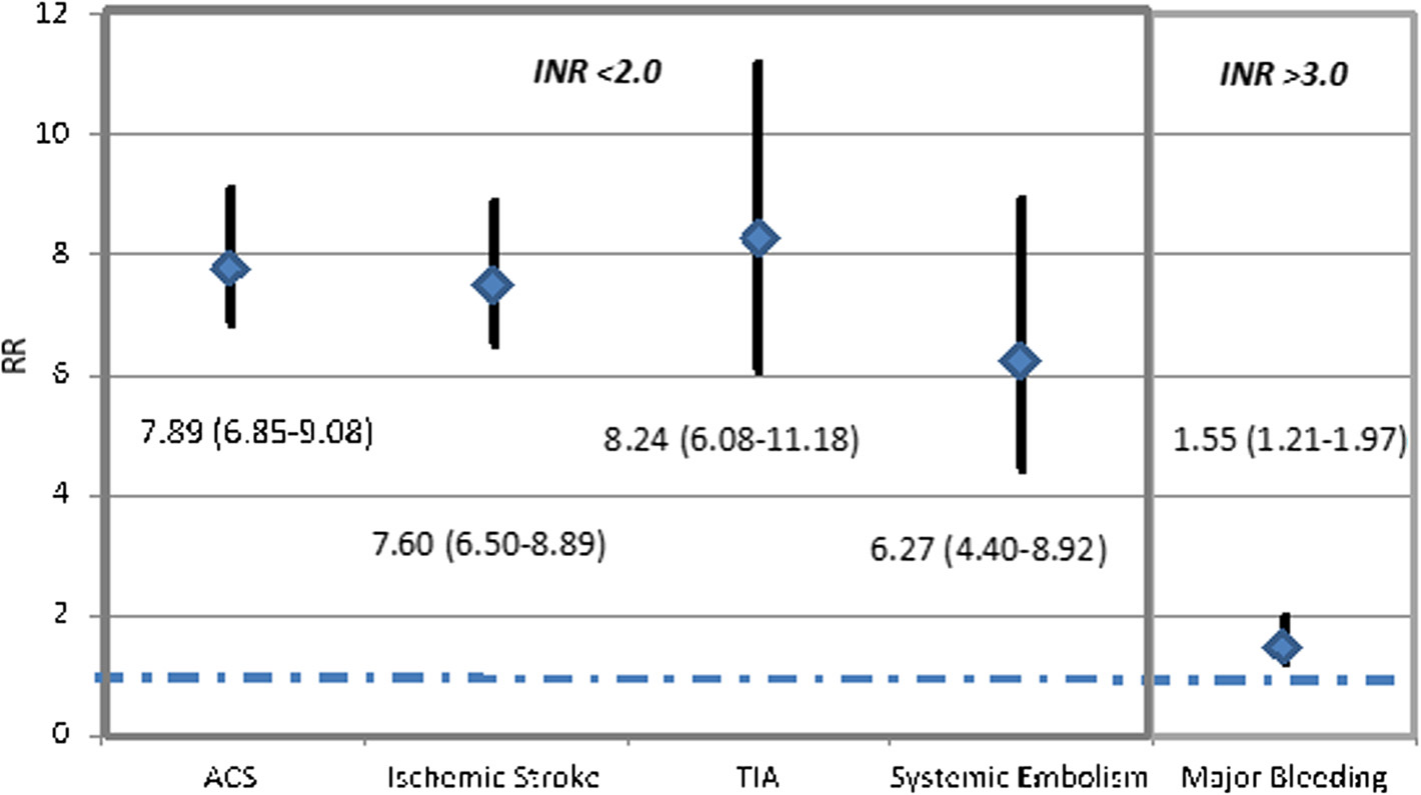
Risks of adverse outcomes for people with INRs <2.0 or >3.0. Adapted from data from an observational study using the Veterans Health Administration dataset [38] showing the relative risk (RR) of adverse thrombotic or embolic events in patients with subtherapeutic INRs versus normal INRs and then major bleeding vents with supertherapeutic INRs versus normal INRs. The diamond represents the actual RR with the line representing the 95 % confidence interval and the blue dashed line representing a RR of 1.0, where the risk of outcomes would have been the same as those with normal INRs

An observational study by Nelson et al, [38] using the Veterans Health Administration (VHA) dataset, explored the relationship between out-of-range INRs and clinical outcomes in 34,346 patients with non-valvular AF (NVAF) who were newly initiated on warfarin therapy. When INR values were below range (<2), patients were much more likely to experience adverse thrombotic or embolic events (Fig. 2). Patients were at an increased risk of major bleeding with both subtherapeutic INR values (RR = 2.58 95%CI: 2.19–3.03) as well as supratherapeutic INR values, (RR = 1.55, 95%CI: 1.21–1.97). All event rates were qualitatively the highest when patients had an INR < 2. While most of these events were stroke associated with sub-therapeutic values, increased bleeding events were also observed. While only speculative (and we could not identify supportive literature) it is possible the increased bleeding associated with sub-therapeutic INRs is due a lag in time between the actual event and the true INR value. This emphasizes the need for close INR monitoring to prevent subtherapeutic warfarin dosing.

Further evidence showed the link between INR instability and clinical events. In meta-analyses that examine the relationship between TTR and the prediction of adverse events, a significant negative relationship has been observed [19]. In patients with AF, 1 thrombotic or major hemorrhagic event per 100 patient-years could be avoided by improving TTR by 7 % or 12 %, respectively [19]. Likewise in patients with VTE, for every 1 % increase in TTR, recurrent thromboembolic events may be reduced by 0.46 % per year and major hemorrhagic events reduced by 0.30 % [17]. Furthermore, in a nested case control analysis of the Atrial fibrillation Clopidogrel Trial with Irbesartan for prevention of Vascular Events (ACTIVE W) study, patients who experienced an ischemic stroke had a TTR 9.5 % lower than those without any ischemic event [39]. The TTR of patients with a major hemorrhage was 7.2 % lower when compared to those without an event, again, suggesting that TTR is a useful predictor for both hemorrhagic and thromboembolic events. Of note, ACTIVE W also found that patients spent a greater amount of time out of range in the 1–2 months preceding a major bleeding event or stroke which suggests even a temporary period out of range can lead to a bleeding event or stroke.

Inability to achieve high TTR in clinical practice is associated with non-persistence and medication discontinuation [40]. In an analysis of longitudinal anticoagulation management records from 15,276 US patients with NVAF, discontinuation of therapy occurred in less than 4 months among patients with unstable INR. Patients who achieved INR stabilization were 10 times more likely to remain on warfarin therapy beyond 1 year. In another observational study using the Symphony Health Solutions’ Patient Transactional Database, patients who were prescribed rivaroxaban had a lower risk of treatment nonpersistence [HR 0.66 (95%CI: 0.60–0.72)] compared to patients who were prescribed warfarin [41]. A similar analysis of the Truven Health Market Scan Research Databases showed comparable findings, that NVAF patients who received rivaroxaban were 46 % less likely to discontinue therapy compared to those receiving warfarin [42]. Continued protection by anticoagulation is particularly important for patients with NVAF, since the risk of stroke is expected to increase with age and additional comorbidities [43].

#### Costs

**Fig. 3:**
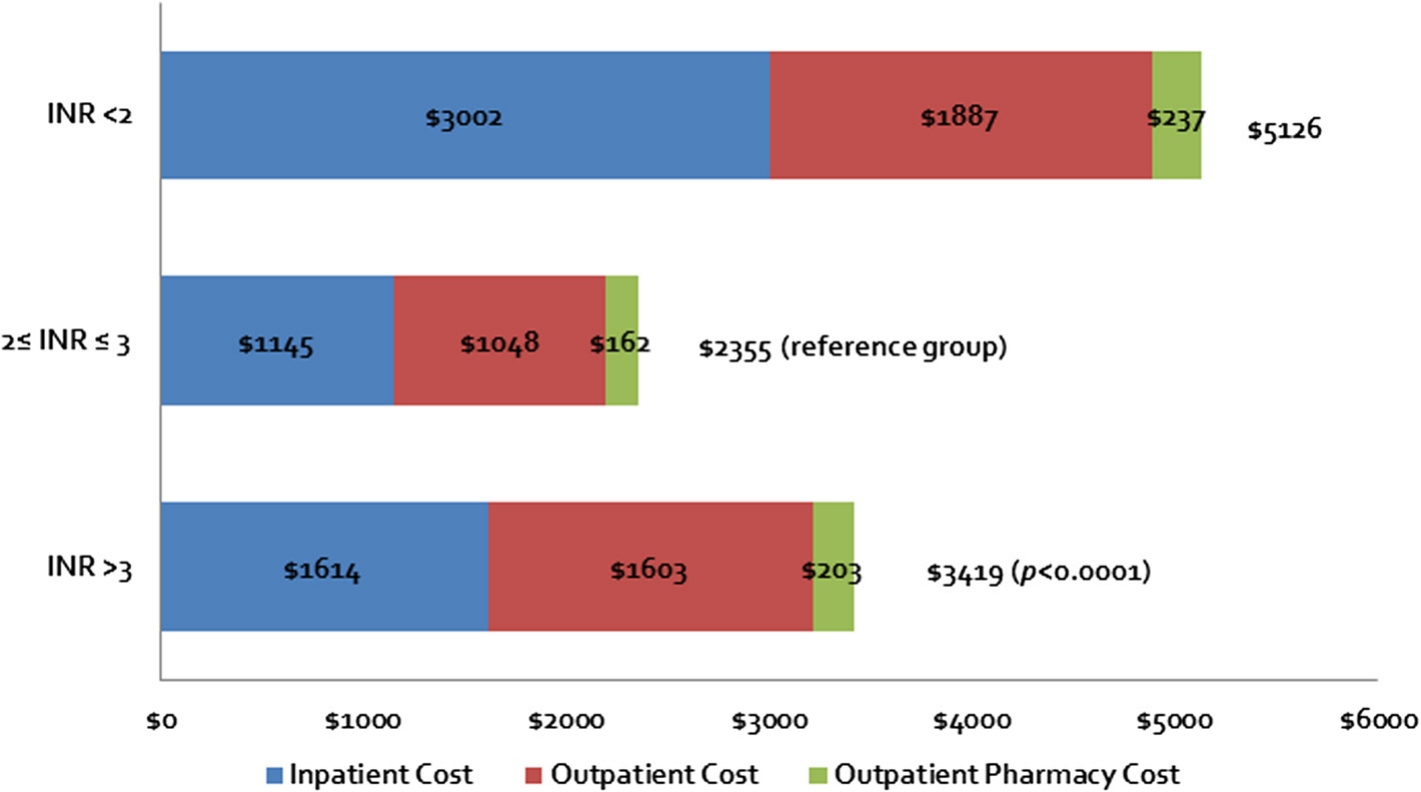
Costs Associated with In Range and Out of Range INRs. Adapted from data from a US Veterans Administration dataset [46] where the total costs are displayed in blue and the constituent costs of inpatient, outpatient, and outpatient pharmacy costs are in red, green, and purple, respectively. The total costs in the therapeutic INR group is significantly lower than those with abnormally low or high INR groups. Note that the highest costs were associated with suboptimal INR values (i.e., INR <2.0)

INR instability was associated with higher healthcare utilization and costs. In an observational study using the Premier Perspective Comparative Hospital Database, hospital length of stay was 5.27 days vs. 4.46 days, leading to significant differences in hospitalization costs ($13,255 vs. $11,993, P < 0.001) [44, 45]. In another comprehensive cost analysis of 23,588 patients with NVAF who were on warfarin for at least 30 days from the US Veteran’s Administration, investigators randomly selected an INR value from a patient and classified it as being below 2, 2–3, or above 3 and then evaluated total direct costs (i.e. inpatient, outpatient medical, and outpatient pharmacy costs) over the next 30 days. Mean direct costs over 30-days after exposure to an INR <2.0, between 2 and 3, and >3.0 were $5126, $2355 and $3419 (Fig. 3) [46]. These findings remained robust in a sensitivity analysis with a more stringent definition of the cohort. The substantial cost difference between in-range and out-of-range time is significant across a broad warfarin population with atrial fibrillation.

The literature suggests that INR instability has important clinical and financial consequences which underscore the need for greater vigilance on achieving INR stability or the use of a novel oral anticoagulant which provides more consistent pharmacologic effects.

### Predicting INR Instability

#### Predictors

Based on data from adjusted meta-regression or multivariate analyses of large datasets, INR stability is known to vary greatly based upon various study- and patient-level factors (Table 3) [15–17, 21, 47, 48]. The use of anticoagulation clinics can positively impact higher TTR attainment but only ~1/3 of VKA patients have access to these advanced services [49]. Therefore, a broad understanding of factors predicting INR instability is beneficial for clinical practice.

**Table 3:** Summative assessment of factors shown to positively or negatively impact INR stability

Factor	Data source	Significant factors the impact INR stability
Poorer INR Stability		
VKA Use in Community Setting vs. Anticoagulation Clinic	Meta-Regression	↓ TTR by 7.1 to 7.2 %
VKA Naïve vs. VKA-Experienced	Meta-Regression	↓ TTR by 5.3 %
Heart Failure	Multivariate Analysis	OR 1.41 for TTR Instability
Diabetes Mellitus	Multivariate Analysis	OR 1.28 for TTR Instability
Stroke History	Multivariate Analysis	OR 1.15 for TTR Instability
Higher CHADS2 Score	Multivariate Analysis	↓ TTR by 7.6 % (SAMe-TT2R2)
Female Gender	Multivariate Analysis	↓ TTR by 6.0 % (SAMe-TT2R2)
Younger Age	Multivariate Analysis	↓ TTR by 20.3 % Age <50 (SAMe-TT2R2)
		↓ TTR by 7.7 % Age 50–60 (SAMe-TT2R2)
Minority Status	Multivariate Analysis	↓ TTR by 18.5 % (SAMe-TT2R2)
Smoking	Multivariate Analysis	↓ TTR by 10.8 % (SAMe-TT2R2)
Amiodarone Use	Multivariate Analysis	↓ TTR by 7.7 % (SAMe-TT2R2)
Better INR Stability		
VKA Self Management vs. No Self Management	Meta-Regression	↑ TTR by 7.0 %
European/United Kingdom Treatment vs. Elsewhere	Meta-Regression	↑ TTR by 9.7 %
Non-Warfarin VKAs vs. Warfarin	Meta-Regression	↑ TTR by 9.2 %
Male Gender	Multivariate Analysis	OR 0.78 for TTR Instability
Hypertension	Multivariate Analysis	OR 0.86 for TTR Instability
Beta-Blocker Use	Multivariate Analysis	↑TTR by 4.8 % (SAMe-TT2R2)
Verapamil Use	Multivariate Analysis	↑ TTR by 6.3 % (SAMe-TT2R2)

*OR* Odds Ratio, *TTR* Time in Therapeutic Range, *VKA* Vitamin K Antagonist

Two of the most extensive studies were conducted by Apostolakis et al. (SAMe-TT2R2) [14] and Rose et al. (VARIA) [48] and provided insight into factors affecting anticoagulation control. Apostolakis and colleagues [14] used data from the 1061 patients in the Atrial Fibrillation Follow-up Investigation of Rhythm Management (AFFIRM) trial to identify clinical factors associated with TTR. Based upon these results, the SAMe-TT2R2 score was derived (and eventually validated) whereby 1 or 2 points are assigned for important patient factors (Table 4). Scores ≥2 were found to be associated with decreased odds of achieving a TTR ≥65 % (previously described as the minimum target TTR) [14].

**Table 4:** SAMe-TT2R2 scoring system and implications

Criteria	One point	Two points	Points
Sex	Female gender		1
Age	Age < 60 years old		1
Medical history	Two or more co-morbidities: • Hypertension • Diabetes • Coronary disease • Peripheral artery disease • Heart failure • Prior stroke • Pulmonary disease • Renal disease • Liver disease		1
Treatment	Treatment with amiodarone		1
Tobacco		Tobacco use in the past 2 years	2
Race		Non-Caucasian race	2
Maximum points			8

The SAMe-TT2R2 score allows an initial patient assessment to discern who is unlikely to achieve a TTR ≥65 %. Patients that score ≥ 2 have reduces odds of achieving TTR ≥65 %. It does not include the risk associated with instability after initiation, only the ability to achieve longer term control [17]

The Veterans AffaiRs study to Improve Anticoagulation (VARIA) [48] used data from over 124,000 veterans receiving warfarin for any indication (55 % AF, 35 % VTE, 10 % other) between 2006 and 2008; and evaluated the effect of various patient characteristics on TTR in those starting warfarin (first 6 months of therapy) and who were experienced (on therapy for >6 months). Like the SAMe-TT2R2 derivation/validation study, female gender, younger age, minority status and co-morbid physical conditions were also found to be associated with lower TTR in VARIA (in both the inception or experienced cohorts) but there were a large number of additional factors which were identified, such as alcohol abuse, number of hospitalizations, and various comorbidities, such as heart failure, diabetes, chronic kidney disease, and others. The VARIA investigators also created a clinical prediction tool but eliminated race because they did not wish to perpetuate disparities in care, eliminated poverty and distance to drive to receive care because they felt it was hard to assess, and eliminated other factors to simplify the model. Their model is available in a downloadable excel spreadsheet from Supplemental Appendix 3S at http://onlinelibrary.wiley.com/doi/10.1111/j.1538-7836.2010.03996.x/full. In addition to the factors in the SAMeTT2R2 score, this tool also assesses the indication for use, total number of chronic medications, substance abuse, mental illnesses, number of hospitalizations, and the general quality of TTR attainment in other patients within that healthcare setting. Even with all of these additional factors, the R-squared value only ranged from 3.2 to 6.8 % suggesting that the much of the variability in TTR is not explained by this model. Furthermore, while the study assessed TTR at therapy initiation and after chronic therapy, the authors stated that the prediction tool is not to be used as a means to assess long term control, and that clinical experience from past VKA therapy is the preferred method [48].

#### Factors that effect INR instability

Two reasons why the clinical prediction tools are inadequate may be related to genetics and adherence to therapy. Patient genotype plays an important role in INR stability [12, 50–52]. At least 30 genes contribute to the anticoagulant effects of VKAs, with one third of the variance in warfarin dosing related to mutations in genes leading to the synthesis of CYP2C9 and vitamin K epoxide reductase (VKORC1) [12, 50–52]. Patients with CYP2C9*2 and CYP2C9*3 polymorphisms have decreased enzymatic activity, metabolize warfarin (and to a lesser extent acenocoumarol) more slowly, have a 1.4- to 3.6-fold increased risk of supratherapeutic INR, and often take longer to achieve stable dosing [53–55]. VKORC1 polymorphisms can result in either a heightened (group A haplotype) or reduced effect (group B haplotype) of warfarin which alters the risk of thromboembolism and bleeding accordingly [12]. Based on this data, the Food and Drug Administration altered the package insert recommending clinicians consider genetic testing before initiating warfarin therapy [56]. However, the cost-effectiveness of this approach is questionable; with economic models suggesting genotyping of patients would cost more than $170,000 per AF patient quality-adjusted life-year (QALY) gained (far above the commonly accepted willingness-to-pay threshold of $50,000 per QALY) [57].

Medication adherence was highlighted as an important variable in VKA INR stability in the American College of Chest Physicians (ACCP) guidelines [12]. Identified predictors of VKA nonadherence include not being married, not having a vehicle for transportation, education levels beyond high school, currently employed, lower levels of mental health functioning, poor cognitive functioning, and greater drug regimen complexity [58, 59]. In addition, studies have identified patient dissatisfaction with care as a cause of medication non-adherence in patients with cardiovascular disease states [60]. This is noteworthy since patient satisfaction with warfarin therapy has been shown to be poor in recent studies of AF [61] and VTE patients [62]. In the above-mentioned studies, poor patient satisfaction in either AF or VTE patients (measure by the Anti-Clot Treatment Scale) was related to the burden and frustration of taking VKAs resulting from fear of bleeding/bruising, diet and alcohol interactions and the perceived hassle of INR monitoring. One of the frequently cited studies evaluating the association between VKA non-adherence and INR control is the International Normalized Ratio Adherence and Genetics (IN-RANGE) Study [63]. IN-RANGE was a prospective cohort study conducted at 3 US anticoagulation clinics and assessed warfarin adherence using a medication electronic monitoring system (MEMS). The study followed 136 patients taking warfarin for a variety of reasons (but predominantly AF and VTE) for a mean of 32-weeks and found that missed warfarin doses (missed MEMS bottle openings) were common, with 92 % of patients missed at least 1 dose, and one-third missed more than 20 % of their doses. A total of 1490 INRs values were collected, with 40 % out of range and 26 % being below range. Upon multivariable regression analysis, researchers found that for every 10 % increase in missed warfarin doses (days without a dose), there was a 14 % increase in the adjusted odds of under-anticoagulation (having an INR < 2.0); and patients who missed >20 % of their doses (missed 20 days of warfarin therapy) had a 2.10-fold (95%CI: 1.48–2.96) increase in their odds of having an INR < 2.0 [63].

Existing research provided good understanding of the drivers behind poor INR control. The research findings indicate that many of the patient factors are not modifiable and are also not sufficiently reliable to predict whether VKA will perform well for a particular patient.

### Modalities to optimize clinical management of VKAs

There are several modalities to improve the clinical management of patients on VKAs including computer assisted dosing and patient self-testing or management. In a randomized study of 13,219 patients conducted in 32 centers around the world, the impact of software program guided VKA therapy was compared with experienced clinician dosing [64]. Unadjusted INR time in range was 67 and 66 % with computer-assisted versus experienced clinician dosing. However, in order to elicit these comparable results, the experienced medical staff randomized to use the computer assisted program provided 11 % of the dosages because the computer failed to provide it and the dose was changed 11 % of the time because the results were felt inaccurate. In addition, the computer-advised appointment intervals were changed by experienced clinicians 34 % of the time. More data is needed to truly determine the impact of computer assisted dosing in less experienced clinicians versus very experienced clinicians/anticoagulation specialists.

Point of care testing by the patient or clinician allow rapid determination of the INR without the need for a centralized laboratory to acquire and analyze the samples. In a meta-analysis of 22 studies, including 4 studied deemed of high methodological quality, the precision and accuracy of the CoaguChek XS, INRatio, ProTime/ProTime3, and Smartcheck INR coagulameter systems were assessed [65]. The CoaguChek system was the most commonly assed and yielded a coefficient of variation that ranged from 1.4 to 5.9 and the concordance ranged between 93 to 100 % showing congruence. The other systems have coefficients of variation ranging from 3.7 to 8.4 and concordance values ranging from 81 to 97 % (with the exception of one trial of the ProTime system where the concordance was only 39 %. In general, point of care testing is accurate and can facilitate patient self-testing (where the patient self-tests the INR but clinician doses) or patient self-management (where the patient self-tests and self-adjusts VKA therapy based on the INR.

In an 8-month open label crossover trial conducted in Canadian primary care offices, patients (*n* = 11, 99 patient months, 122 INR determinations) underwent patient self-management or physician management for 4 months [66]. Patients were trained and given an algorithm to follow that specified the new dose and the timeframe for which to reassess the INR. The mean proportion of INR values in therapeutic range among subjects in the PSM and physician-management groups was 82 and 80 %, respectively (*p* = 0.82). Ten of the 11 patients preferred PSM to physician management and elected to continue with this strategy after study completion (*P* = .001). No calls or visits were made to the physician regarding dose adjustment during the patient self-management period. There were no episodes of major bleeding or thromboembolic events. Studies like this are promising but preliminary and it is unclear whether this is an effective therapy for highly motivated and intelligent patients or patients with health disparities or care barriers.

## Conclusions

While experts recommend that patients spend at least 65 % of their TTR, this is seldom achieved or sustained over time. A more common pattern – based on the literature – is that there is high variability, and even the patients who achieve target INR range do not remain in this target range for long. Patients are more likely to have INR below the therapeutic range, exposing them to significant risk of adverse clinical events. Not achieving a high TTR can result in thrombotic and major bleeding events, inability to remain on therapy, and higher cost of care. To avoid the adverse consequences of unstable INR, careful evaluation of patients prior to initiating therapy is important. Clinical prediction tools are available, though they can only explain <10 % of the variance behind poor INR control because of the complex drivers of genetic variation and patient medication adherence. Clinicians caring for patients who require anticoagulation are encouraged to apply intensified diligence in INR management when using VKAs.

## Abbreviations

ACCP, American College of Chest Physicians; ACTIVE W, Atrial fibrillation Clopidogrel Trial with Irbesartan for prevention of Vascular Events; AF, atrial fibrillation; CI, confidence interval; DVT, deep vein thrombosis; GARFIELD, Global Anticoagulant Registry in the FIELD; ICER, incremental cost-effectiveness ratio; ICH, intracerebral hemorrhage; INR, international normalized ratio; IN-RANGE, International Normalized Ratio Adherence and Genetics; MEMS, medication electronic monitoring system; NOAC, novel oral anticoagulant; PE, pulmonary embolism; PINRR, proportion of INR measurements in range; QALY, quality-adjusted life-year; RCT, randomized controlled trial; TTR, time in therapeutic range; UK, United Kingdom; VARIA, Veterans AffaiRs Study to Improve Anticoagulation; VKA, vitamin K antagonist; VKOR, vitamin K epoxide reductase
